# Metabolic reprogramming in skeletal cell differentiation

**DOI:** 10.1038/s41413-024-00374-0

**Published:** 2024-10-11

**Authors:** Joshua C. Bertels, Guangxu He, Fanxin Long

**Affiliations:** 1 Department of Surgery, Translational Research Program in Pediatric Orthopedics, The Children’s Hospital of Philadelphia, Philadelphia, PA USA; 2 Department of Orthopedics, The Second Xiangya Hospital, Changsha, Hunan China; 3 Department of Orthopedic Surgery, University of Pennsylvania, Philadelphia, PA USA

**Keywords:** Bone, Homeostasis

## Abstract

The human skeleton is a multifunctional organ made up of multiple cell types working in concert to maintain bone and mineral homeostasis and to perform critical mechanical and endocrine functions. From the beginning steps of chondrogenesis that prefigures most of the skeleton, to the rapid bone accrual during skeletal growth, followed by bone remodeling of the mature skeleton, cell differentiation is integral to skeletal health. While growth factors and nuclear proteins that influence skeletal cell differentiation have been extensively studied, the role of cellular metabolism is just beginning to be uncovered. Besides energy production, metabolic pathways have been shown to exert epigenetic regulation via key metabolites to influence cell fate in both cancerous and normal tissues. In this review, we will assess the role of growth factors and transcription factors in reprogramming cellular metabolism to meet the energetic and biosynthetic needs of chondrocytes, osteoblasts, or osteoclasts. We will also summarize the emerging evidence linking metabolic changes to epigenetic modifications during skeletal cell differentiation.

## Introduction

Skeletal diseases represent a significant burden on human health worldwide. Many factors, ranging from aging to estrogen deficiency, have been shown to alter the efficiency of bone forming osteoblasts and bone resorbing osteoclasts, resulting in osteopenia or osteoporosis. Increasingly, systemic metabolic disorders such as diabetes mellitus have been linked with increased bone fragility in human patients.^1–5^ The bone deficiency is partly attributed to dysregulation of energy metabolism in osteoblasts.^6^ Diabetes has also been identified as an independent predictor for severe osteoarthritis (OA), among many other driving factors such as genetics and aging.^7,8^ In addition, obesity is linked to bone weakening due to increased bone resorption over formation.^9^ Likewise, hyperlipidemia associated with excessive levels of cholesterol may cause osteoporosis and osteoarthritis.^10^ Thus, numerous genetic and environmental factors influence skeletal tissue health and diseases.

To understand the pathogenesis of skeletal diseases as a basis for developing safe and effective therapies, it is paramount to elucidate the mechanisms controlling the differentiation and function of the key cell types, including chondrocytes, osteoblasts, and osteoclasts. Extensive studies in the field have focused on transcription factors and intercellular signals that influence skeletal cell differentiation and function. More recently, much has been learnt about not only the metabolic features of each skeletal cell type, but also the mechanisms for differentiation signals to reprogram cellular metabolism. Notably, metabolic reprogramming not only fulfills new energetic and biosynthetic needs but also influences gene expression via epigenetic modifications.

In this review, we highlight the molecular and metabolic regulation of osteoblasts, osteoclasts and chondrocytes. For each cell type, we will first briefly describe the transcription and growth factors regulating its differentiation. We will then focus on the use of various energy substrates by each cell type, and how the metabolic changes intersect with the transcription or growth factors during differentiation. Lastly, we will discuss the emerging connection between metabolites and epigenetic regulation of the differentiation process.

## Overview of mammalian cell metabolism

### Glucose metabolism

**Fig. 1:**
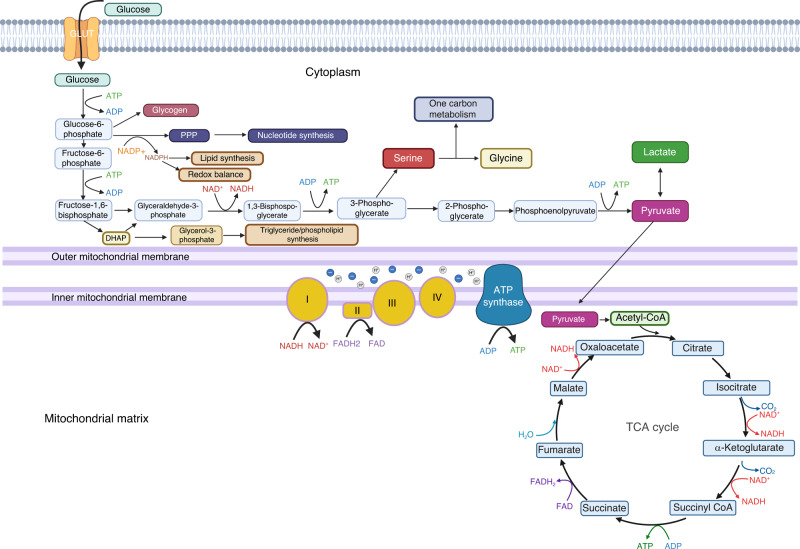
A diagram for glucose metabolism in mammalian cells. Core glycolysis pathway along with several side branches are depicted. The end-product of glycolysis pyruvate can enter the mitochondria to fuel cellular respiration or converted to lactate in the cytosol

Glucose is a major energy substrate for most mammalian cells. Glucose, upon transported across the plasma membrane into the cell through the GLUT family of transporters in most cell types, is then phosphorylated into glucose-6-phosphate (G6P) by hexokinase to prevent it from leaving the cell^11,12^ (Fig. 1). Apart from entering the pentose phosphate pathway for nucleotide synthesis, or being converted to glycogen, G6P is mainly catabolized through the glycolysis pathway.^12^ Through a multi-step process, glucose is metabolized in the cytosol until it is converted into pyruvate, netting two adenosine triphosphate (ATP) molecules, and reducing two nicotinamide adenine dinucleotide (NAD^+^) molecules into nicotinamide adenine dinucleotide hydrogen (NADH) per glucose molecule. In the process, dihydroxyacetone phosphate (DHAP), can enter a side pathway to form glycerol as the backbone of phospholipids and triglycerides. In addition, 3-glycerophosphate (3PG) can enter the serine synthesis pathway to generate serine and glycine, as well as feeding the one-carbon metabolism pathway to produce substrates necessary for epigenetic regulation and biosynthesis. Thus, besides energy and pyruvate production, glycolysis generates intermediary metabolites that enter side pathways with distinct functions.

Once produced from glycolysis, pyruvate can either be transported into the mitochondria through the mitochondrial pyruvate carrier and converted to acetyl co-enzyme A (acetyl-CoA) by pyruvate dehydrogenase (PDH) or remain in the cytosol to become lactate through lactate dehydrogenase (LDH). If the former is performed, acetyl-CoA will enter the tricarboxylic acid (TCA) cycle, generating more molecules of NADH and flavin adenine dinucleotide (FADH_2_), which will then be used in the electron transport chain (ETC) to generate 30-plus molecules of ATP via oxidative phosphorylation. During this process, reactive oxygen species (ROS) are generated and play a role in certain signaling pathways, yet must be moderated to prevent oxidative stress and damages.^13^ Whereas ATP extraction from pyruvate in the mitochondria requires oxygen, pyruvate conversion to lactate in the cytosol does not and can occur in both aerobic and anaerobic conditions. When glucose is predominantly converted to lactate in aerobic conditions, the process is often referred to as aerobic glycolysis, or the Warburg effect as it was originally described by Otto Warburg in cancer cells.^14–16^ The metabolic fate of glucose through the various pathways is highly dependent on the energy and biosynthetic needs of each cell.

### Amino acid metabolism

**Fig. 2:**
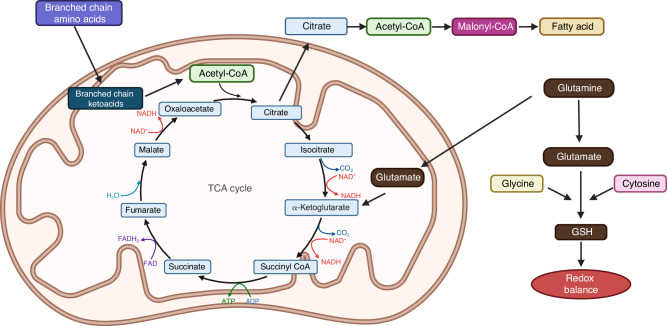
Examples of amino acid catabolism in mammalian cells. Multiple amino acids can enter the TCA cycle via various intermediates (e.g., oxaloacetate, fumarate) but are not depicted here for simplicity

Besides glucose, amino acids also contribute to energy production via mitochondrial oxidative phosphorylation beyond their role as direct building blocks in protein synthesis.^17,18^ For example, glutamine produces alpha-ketoglutarate (α-KG) in the mitochondria to replenish the TCA cycle metabolites, whereas branched amino acids (leucine, isoleucine, and valine) generate acetyl-CoA to fuel the TCA cycle (Fig. 2). Glutamine also plays a vital role in the synthesis of glutathione (GSH), a potent cellular antioxidant. In addition, methionine, together with acetyl-CoA derived from branched chain amino acids or glutamine, provide essential substrates for histone and DNA modifications, thus regulating gene expression.^17^

### Fatty acid metabolism

**Fig. 3:**
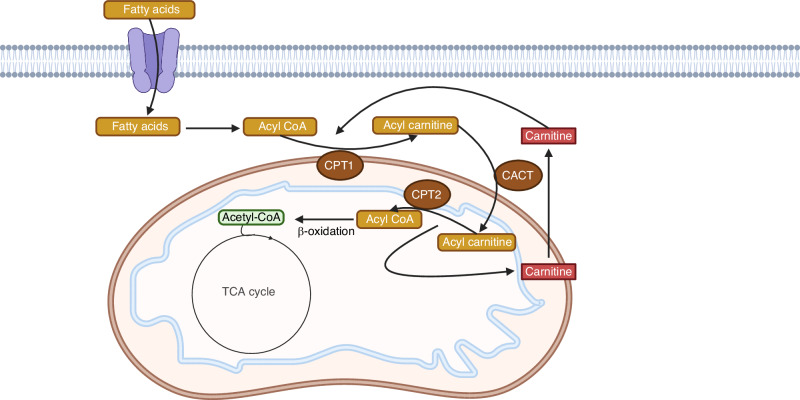
A diagram for long-chain fatty acid oxidation in the mitochondria. CPT1/2: carnitine palmitoyltransferase 1/2. CACT: carnitine acylcarnitine translocase

Fatty acids are a major energy source by fueling the TCA cycle following β-oxidation.^19^ Fatty acids are transported into the cytosol of cells via proteins such as CD36 and plasma membrane fatty acid binding protein (FABPpm, also known as GOT2). Different from short and medium-chain fatty acids that can enter the mitochondria directly, long-chain fatty acids are first converted to acyl-coA in the cytosol and must be transported by the carnitine shuttle through a multi-step process (Fig. 3). In this process, carnitine palmitoyltransferase 1 (CPT1), an outer mitochondrial-membrane acyltransferase, is responsible for the conversion of acyl-CoA into acylcarnitine. Carnitine acylcarnitine translocase (CACT) then transfers acylcarnitine into the mitochondria in exchange for free carnitine, before CPT2 reverts acylcarnitine back into acyl-CoA. Once inside the mitochondria, acyl-CoA it is then degraded into acetyl-CoA sequentially via β-oxidation to enter the TCA cycle.^19^

### Metabo-epigenetic regulation of cell fate and activity

**Fig. 4:**
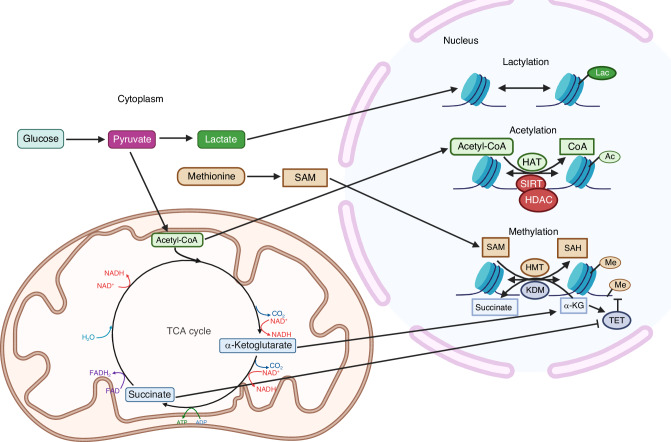
Depiction of metabo-epigenetic regulation. SAM: S-adenosyl methionine. SAH: S-adenosyl homocysteine. α-KG: alpha-ketoglutarate. HMT: histone methyltransferase. KDM: lysine demethylase. TET: ten-eleven translocation. SIRT: sirtuin. HDAC: histone deacetylase. HAT: histone acetyltransferase

The interplay of cellular metabolism and gene expression has become an active area of research. It is now well accepted that changing levels of certain metabolites can alter histone and DNA modifications resulting in changes in gene expression.^20^ Herein we refer to the phenomenon as metabo-epigenetic regulation. Multiple metabolites have been shown to provide modifications to the epigenome, which in turn reprogram the transcriptome.^21,22^ Acetyl-CoA and S-adenosyl-L-methionine (SAM) are necessary co-substrates for the acetylation and methylation of DNA and histones, which are carried through histone acetyltransferases (HATs) and methyltransferases (MTs), respectively (Fig. 4). In addition, ten-eleven translocation (TET) and jumonji lysine demethylases (Jmj-KDMs) use α-ketoglutarate as a co-factor for demethylation of DNA and histones, respectively, whereas sirtuins act as NAD^+^-dependent histone deacetylases.^23^ Recently, lactate was found to modify histones via lactylation.^24^ This modification has been implicated in tumorigenesis and cell fate changes related to myopia pathogenesis.^25–27^ Although there are ample examples of metabo-epigenetic regulation in other cell types, evidence in skeletal cell differentiation is just emerging.

## Chondrocytes

### Growth factors and nuclear regulators in chondrocyte differentiation

**Fig. 5:**
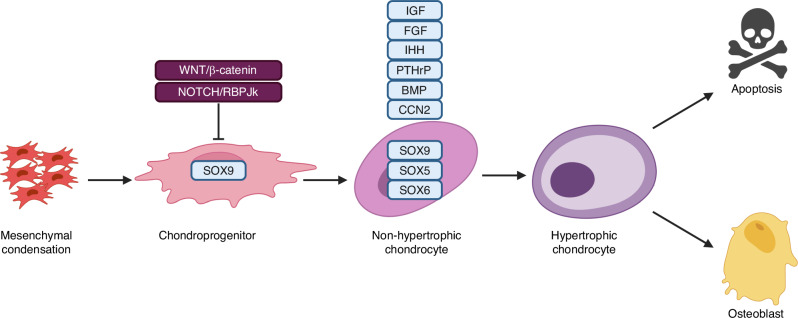
A summary of major transcription factors and growth factors regulating chondrocyte differentiation and function. Information is derived from studies of chondrocytes during endochondral skeletal development. For simplicity, non-hypertrophic chondrocyte encompasses various stages before the onset of hypertrophy. Blocked arrow denotes inhibition. See text for details and references

Chondrocytes are cartilage resident cells responsible for the deposition of the cartilage extracellular matrix (ECM) composed of mainly collagen II and the predominant proteoglycan aggrecan. During embryogenesis, cartilage provides the template for most of the skeleton which is formed through endochondral ossification.^28^ The initiation of chondrogenesis begins with the expression of transcription factor Sry-related high-mobility-group box 9 (SOX9) following mesenchymal condensation^29^ (Fig. 5). Activation of SOX9 appears to be partly mediated by HIF1α in response to hypoxia in the mesenchymal condensations.^30^ In vitro live imaging experiments showed that SOX9 is likely dispensable for the initial condensation of mesenchymal cells.^31^ Consistent with the view, mouse genetic studies showed that a genetic block of bone morphogenic proteins (BMP) signaling in the early limb mesenchyme prevents mesenchymal condensation independent of SOX9 expression.^32^ However, conditional deletion of SOX9 in early limb mesenchyme with Prx1-cre led to the absence of mesenchymal condensations in mouse embryos, indicating that SOX9 is likely required for maintaining the mesenchymal condensations besides its later function in chondrogenesis.^33^ Following SOX9 activation, L-SOX5 and 6 are also expressed in the chondrogenic cells and are required for completion of proper chondrocyte differentiation as indicated by activation of COL2A1 (encoding collagen II) and ACAN (encoding aggrecan) expression.^33,34^ In cartilage destined to be replaced by bone via endochondral ossification, chondrocytes, typically organized as morphologically discrete domains in growth plates, progressively mature and eventually undergo hypertrophy characterized by COL10A1 (encoding collagen X) expression. Whereas some hypertrophic chondrocytes undergo apoptosis, others have been shown in mice to become osteoblasts contributing especially to trabecular bone at the embryonic and neonatal stages.^35–38^

A number of growth factors have been implicated in chondrocyte differentiation and maturation. FGF signaling has been shown to stimulate Sox9 expression via the MAPK pathway in both chondrocytes and in undifferentiated mesenchymal cells.^39^ Besides their apparent role in inducing mesenchymal condensations as discussed above, BMP are critical for completion of the subsequent chondrogenic program as conditional deletion of BMP type I receptors BMPR1A and BMPR1B, or the transcriptional mediators SMAD1 and SMAD5 with Col2a1-Cre caused severe chondrodysplasia.^40,41^ Related to BMP, transforming growth factor β (TGF-β) also plays multiple roles in cartilage development. Contrary to early suggestions from in vitro experiments with added TGF-β protein, TGF-β signaling is dispensable for chondrogenesis in vivo as deletion of the obligatory receptor Tgfbr2 in the limb mesenchyme did not affect the initial cartilage anlagen.^42^ TGF-β signaling however is required for proper chondrocyte proliferation as well as the progression to terminal hypertrophy, besides a prominent role in synovial joint development.^42,43^ Following establishment of the growth plate, Indian hedgehog (IHH) and parathyroid hormone related protein (PTHrP) are essential growth factors controlling the progression of immature chondrocytes to their hypertrophic stage, and eventual endochondral ossification.^44–46^

The insulin-like growth factors, IGF1 and IGF2, are important regulators of both prenatal and postnatal body growth.^47^ Genetic studies have shown that IGF signaling through IGF1R functions in parallel with IHH to regulate chondrocyte proliferation and cartilage growth during embryonic development.^48^ After birth, IGF2 has also been shown to regulate skeletal growth in both humans and mice.^49,50^ The IGF2 knockout mice exhibited a disproportionally large hypertrophic zone in the growth plate of early postnatal mice, indicating an important role of IGF2 in controlling the progression of hypertrophy.^50^

Connective tissue growth factor (CTGF/CCN2) is a secreted protein containing several domains that mediate interactions with growth factors, integrins and extracellular matrix components.^51^ CCN2 deletion impaired chondrocyte proliferation, reduced cartilage matrix production and retarded hypertrophy, resulting in skeletal dysmorphisms.^52,53^ Interestingly, the loss of CCN2 caused an upregulation of another family member CCN3 in chondrocytes which functioned opposite to CCN2 and likely contributed to the cartilage phenotype in CCN2-null mice.^53^

Besides the positive regulators, Wnt/β-catenin signaling has been shown to inhibit chondrocyte differentiation.^54^ In chick embryos or limb bud cultures, Wnt4 overexpression blocked the initiation of chondrogenesis but promoted terminal differentiation of chondrocytes.^55,56^ In contrast, Wnt5a or Wnt5b promoted chondrogenesis while inhibiting chondrocyte terminal differentiation, likely through β-catenin independent mechanisms.^55,56^ Genetic studies of β-catenin in mouse embryos confirmed that β-catenin suppresses chondrogenesis in the limb mesenchyme.^57,58^ Biochemically, β-catenin has been shown to suppress overt chondrocyte differentiation via physical interaction with Sox9.^59^ Thus, both genetic and biochemical evidence supports Wnt/β-catenin signaling suppresses chondrocyte differentiation.

Notch signaling has also been implicated in suppressing chondrogenesis from mesenchymal progenitors. Loss- or gain-of-function studies of Notch/RBPjk signaling showed that chondrogenesis was either enhanced or impaired, respectively, in mouse embryos.^60^ The anti-chondrogenic effect of Notch/RBPjk signaling could potentially be due to the suppression of Sox9 transcription.^61^ Moreover, Notch signaling suppressed chondrocyte proliferation in the growth plate apparently independent of RBPjk while it also promoted both the onset and terminal progression of chondrocyte hypertrophy in a RBPjk dependent manner.^62^ The studies therefore highlight the multiple roles and mechanisms of Notch signaling in regulating chondrocyte development.

### Chondrocyte metabolic regulation

Production of substantial amounts of cartilage extracellular matrix requires a significant energy supply in chondrocytes. Glycolysis plays a vital role in fulfilling the bioenergetic need in those cells.^12^ Knockout studies have demonstrated that GLUT1, the predominant glucose transporter in chondrocytes, is critical for proper proliferation, matrix production and hypertrophy of growth plate chondrocytes during embryonic development.^63^ As discussed earlier, glycolysis provides not only energy but also building blocks necessary for biosynthesis. The glycolytic intermediate 3-phosphoglycerate (3PG) can be diverted to the serine biosynthesis pathway, contributing to one-carbon metabolism to produce purines and thymidine necessary for DNA replication. Knockout of phosphoglycerate dehydrogenase (PHGDH), the key enzyme for directing 3PG for serine biosynthesis, impaired proliferation of growth plate chondrocytes, presumably due to insufficient nucleotides.^64^ Thus, glucose metabolism is critical for fulfilling both bioenergetic and biosynthetic needs in chondrocytes.

Like growth plate cartilage, articular cartilage of the joints also requires significant metabolic input from glucose. Multiple glucose transporters including GLUT1 and GLUT3 are expressed in articular chondrocytes and regulated by both cartilage anabolic and catabolic signals.^65–67^ Genetic studies have demonstrated that GLUT1 is required for articular chondrocyte survival and joint cartilage maintenance in adult mice particularly in the context of surgery-induced osteoarthritis.^68,69^ Furthermore, forced expression of GLUT1 was sufficient to ameliorate joint cartilage loss in the surgery-induced osteoarthritis mouse model, supporting impaired glucose metabolism as a driver in osteoarthritis progression.^68^

**Fig. 6:**
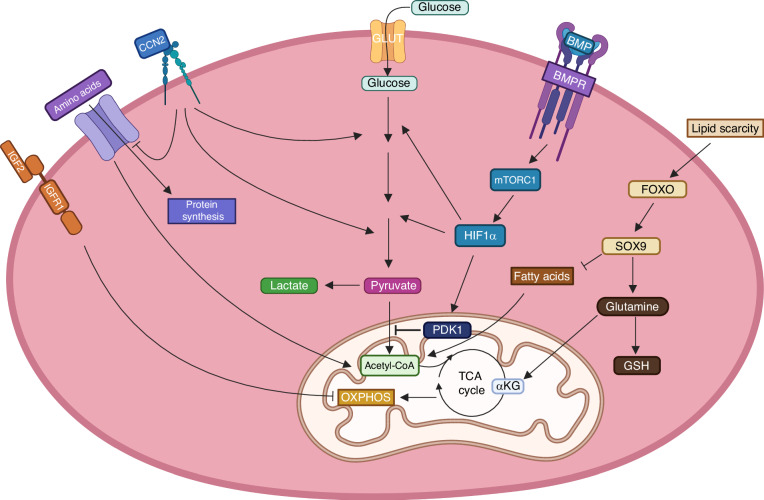
Metabolic regulation of chondrocyte differentiation by transcription factors and growth factors. Blocked arrow denotes inhibition whereas pointed arrow indicates stimulation. PDK1: pyruvate dehydrogenase kinase 1. GSH: reduced glutathione. α-KG: alpha-ketoglutarate

HIF1α is a transcription factor that is stabilized in response to hypoxia and directly activates many downstream target genes to allow for the adaptive responses.^70^ HIF1α is well known to stimulate expression of most genes in the core glycolysis pathway along with PDH kinase that restricts pyruvate from fueling the TCA cycle, thus reducing oxygen consumption from mitochondrial respiration^71,72^ (Fig. 6). Chondrocytes are unique among skeletal cells as they reside in the avascular cartilage and therefore have limited access to oxygen. Tissue-specific deletion studies in mouse embryos have shown that HIF1α is essential for the survival of hypoxic chondrocytes residing at the core of growth plate cartilage.^73^ A critical aspect of the protective role is mediated by active suppression of mitochondrial respiration as HIF1α is dispensable for chondrocyte survival when mitochondrial transcription factor A (TFAM) is simultaneously deleted.^74^

Although glycolysis is the major bioenergetic pathway in growth plate chondrocytes, a role for TCA metabolism and mitochondrial respiration has also been uncovered. Sustained HIF1α signaling due to deletion of PHD2, a protein that modifies HIF1α for degradation, caused an energy deficit in growth plate chondrocytes that could not be fully compensated by increased glycolysis, resulting in reduced cell proliferation and matrix production.^75^ In addition, reduced acetyl-CoA levels due to inhibition of PDH activity has been shown to delay the maturation of growth plate chondrocytes in SIK3 knockout mice.^76^ Similarly, deletion of TFAM delayed hypertrophy without affecting chondrocyte proliferation or survival in mouse embryonic growth plate.^74^ In keeping with the in vivo studies, cultured hypertrophic chondrocytes exhibited an increase in OXPHOS and reduced glycolysis when compared to proliferating chondrocytes, whereas inhibition of OXPHOS suppressed chondrocyte hypertrophy.^77^ Thus, proper coordination between glycolysis and mitochondrial respiration is necessary to support the full spectrum of chondrocyte activity at different developmental stages.

Several studies have highlighted the roles of amino acid metabolism in chondrocytes. As mentioned earlier, de novo serine synthesis through phosphoglycerate dehydrogenase (PHGDH) is critical for supplying adequate levels of intracellular serine necessary for nucleotide synthesis during chondrocyte proliferation.^64^ In addition, in vitro studies have demonstrated the positive effects of proline, lysine and glycine on collagen II production by chondrocytes.^78^ Interestingly, unlike proline and lysine, glycine maintained the positive effect even when applied at high concentrations beyond its physiological range. Whether or not this reflected a potential role beyond being a building block of collagen II remains to be elucidated. The branched-chain amino acid leucine supported chondrocyte proliferation and hypertrophy in both mTOR-dependent and -independent mechanisms, whereas glutamine was necessary for adequate protein synthesis in chondrocyte cultures.^79,80^ Finally, mouse genetic studies support critical roles for glutamine metabolism in multiple functions of chondrocytes including matrix gene expression, proliferation and redox balance, as further discussed below.^81^

SOX9, the master transcription factor for chondrogenesis, has direct effects on the metabolism of chondrocytes (Fig. 6). SOX9 increases glutamine consumption and levels of glutaminase 1 (GLS1) to support various chondrocytes properties via several mechanisms, these including histone acetylation through acetyl-CoA synthesis, aspartate formation for protein synthesis as well as glutathione production for protection against harmful ROS.^81^ SOX9 has also been linked with changes in fatty acid metabolism during chondrogenesis. Lipid scarcity in the avascular environment increased SOX9 expression via activation of FOXO whereas SOX9 in turn suppresses fatty acid oxidation.^82^ Thus, besides inducing the expression of chondrocyte identity genes, SOX9 simultaneously alters cellular metabolism to meet the specific bioenergetic and biosynthetic needs in the avascular environment during chondrogenesis.

The roles of chondrogenic growth factors in metabolic regulation are just beginning to be uncovered (Fig. 6). Genetic deletion of BMPR1A has linked the role of BMP signaling in chondrocyte proliferation and hypertrophy with the regulation of GLUT1 expression in growth plate chondrocytes.^63^ Mechanistically, BMP2 signaling boosts glycolysis in growth plate chondrocytes in vitro through activation of the mTORC1-HIF1α pathway.^63^ In contrast, in human articular chondrocytes isolated from osteoarthritis patients, BMP2 increased OXPHOS instead of glycolysis whereas TGFβ1 exhibited the opposite effects.^83^ Although it is not clear whether osteoarthritis skews the metabolic response of chondrocytes to the growth factors, those results seem to indicate that BMP/TGFβ signaling regulates cellular metabolism differently in growth plate versus articular chondrocytes.

CCN2, which stimulates chondrocyte proliferation, proteoglycan synthesis and chondrocyte hypertrophy in the growth plate, has been shown to increase energy production from glycolysis through upregulation of the glycolysis genes PGK1, PGAM1 and ENO1^84,85^ (Fig. 6). Moreover, increased amino acid consumption was observed in the CCN2-deficicient chondrocytes, in part reflecting a compensatory mechanism for aerobic energy production in response to impaired glycolysis.^85^ Finally, the role of IGF2 in stimulating cartilage growth in early postnatal mice has been linked to its role in modulating glucose metabolism in chondrocytes, likely by suppressing OXPHOS to control the progression of hypertrophy.^50,77^

### Metabo-epigenetic regulation of chondrogenesis

Epigenetic regulation by metabolites during chondrogenesis is just beginning to be unraveled. SOX9 increased glutamine consumption which in turn stimulated chondrogenic gene expression through upregulation of histone acetylation.^81^ The increased histone acetylation appeared to be downstream of acetyl-CoA synthesis from glutamine-derived citrate.^81^

## Osteoblasts

### Growth factors and nuclear regulators in osteoblast differentiation

**Fig. 7:**
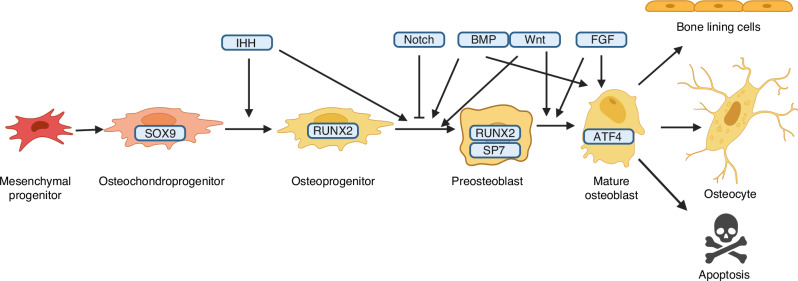
Key transcription factors and growth factors regulating osteoblast differentiation and function. For simplicity, interactions between BMP and WNT or ACTIVIN signaling is not depicted (see text). Blocked arrow denotes inhibition whereas pointed arrow indicates stimulation. See text for details and references

Osteoblast differentiation is driven by stepwise activation of multiple transcription factors^86^ (Fig. 7). The earliest osteoblastogenic progenitors, like chondrogenic cells, express SOX9.^87^ Subsequent activation of RUNX2, a runt domain-containing transcription factor, is indispensable for osteoblast differentiation.^88,89^ Consistent with its central role in osteoblast differentiation, RUNX2 itself is regulated by numerous other nuclear factors that modulate RUNX2 expression or activity either positively or negatively.^90^ Following the initiation and commitment stage, osteoblast differentiation requires activation of OSX (official name SP7), a zinc finger-containing transcription factor, whereas activating transcription factor 4 (ATF4) promotes osteoblast activity in the more mature cells.^91,92^ Mature osteoblasts subsequently become osteocytes entombed in the bone matrix, or bone lining cells on the bone surface, or undergoing apoptosis, but the mechanisms responsible for the fate choices are not known at present.^93^

The growth factors that influence osteoblast differentiation have also been extensively studied (Fig. 7). Indian Hedgehog (IHH), a member of the Hedgehog family, is produced by prehypertrophic and early hypertrophic chondrocytes in the growth plate, and its direct signaling to the progenitor cells in both perichondrium and the primary spongiosa is required for osteoblastogenesis in the developing endochondral skeleton of mouse embryos.^45,94^ The osteogenic role of IHH requires both de-repression of the GLI3 transcription repressor and the activator function of GLI2.^95,96^ Whereas GLI3 de-repression is sufficient to induce RUNX2 expression in the progenitors, RUNX2 alone cannot replace IHH function in osteoblast differentiation.^96,97^ Indeed, additional activation of GLI2 is required for the transition to the OSX+ stage and completion of the osteoblast differentiation program.^95^ Inducible deletion of SMO, the obligatory cell-surface signal transducer of Hedgehog signaling, in juvenile mice, impaired trabecular osteoblast differentiation in long bones.^98^ The data therefore support critical roles of Hedgehog signaling in both periosteal and trabecular osteoblast differentiation during fetal as well as postnatal development of endochondral bones.

BMP signaling pathways have been shown to play important roles in osteoblast differentiation. Knockout studies showed that a critical threshold level of BMP2 and BMP4 is required for the transition of Runx2^+^ to Runx^+^ OSX^+^ cells.^99^ (Fig. 7) Aside from SMAD-mediated signaling, BMP also activates mTORC1 signaling to augment the anabolic activity in osteoblast-lineage cells.^100,101^ Unexpectedly, deletion of one of the BMP type I receptors BMPR1A or ACVR1 in osteoblast-lineage cells increased trabecular bone mass at least partly due to reduced expression of the WNT antagonist SOST.^100,102–106^ Deletion of one of the type II BMP receptors BMPR2 also increased bone formation likely due to diminished activin signaling.^107^ Thus, the mechanisms for BMPs and other members of the TGFβ superfamily to regulate osteoblast differentiation are complex and remain to be fully elucidated.

WNT signaling has been extensively studied for its role in osteoblast differentiation and activity.^108^ In particular, deletion of β-catenin, a central mediator of WNT signaling, in skeletogenic progenitors abolishes mature osteoblast production as β-catenin is required for the progression of Runx2^+^ to Runx2^+^ OSX^+^ cells and then to mature osteoblasts.^57,58,109,110^ (Fig. 7) The loss of osteoblasts was recapitulated in mouse embryos lacking the WNT receptors LRP5 and LRP6, further supporting that β-catenin functions in the WNT signaling pathway to control osteoblast differentiation.^111^ Besides β-catenin, PKC and mTOR activation by WNT proteins have also been shown to stimulate osteoblast differentiation and function.^112–114^

Mouse genetic studies have also uncovered important roles for FGF signaling in the osteoblast lineage.^115^ (Fig. 7) FGF18 deficient mouse embryos exhibited defects in osteoblast maturation despite normal RUNX2 expression, whereas loss of FGF2 reduced bone formation in adult mice.^116,117^ Deletion studies of the receptors FGFR1 or FGFR2 in osteogenic progenitors support their stimulatory role in osteoblast differentiation and function, whereas FGFR3 appears to be required for optimal bone mineralization.^118–120^ Remarkably, conditional deletion of FGFR1 or both FGFR1 and FGFR2 in mature osteoblasts caused osteocyte death and bone mass overgrowth secondary to increased WNT signaling.^121^ Thus, FGF proteins through various receptors regulates multiple aspects of osteoblast differentiation and function.

NOTCH signaling mediates communication between neighboring cells through cell-cell contact and has been implicated in osteoblast differentiation. Contrary to the pathways above, NOTCH signaling suppresses osteoblastogenesis as deletion of NOTCH1 and NOTCH2 or the critical transcription factor RBPJk in the embryonic limb mesenchyme enhanced osteoblast differentiation at the expense of bone marrow mesenchymal progenitors.^122,123^ The suppression occurs prior to the OSX^+^ stage and is partly through the inhibition of RUNX2 activity.^122,123^ (Fig. 7) Consistent with the loss-of-function studies, NOTCH overactivation suppressed osteoblast differentiation from early progenitors.^124^ However, when NOTCH was overactivated at a later stage it caused overproduction of immature osteoblasts like those observed in osteosarcoma.^125,126^ Thus, NOTCH signaling exerts stage-specific functions in osteoblast lineage cells.

### Osteoblast metabolic regulation

Several historical studies have shown that bone explants and osteoblasts in culture rapidly consume glucose and converts it to lactate even when maintained with atmospheric levels of oxygen.^127–131^ Recent use of advanced techniques such as Seahorse and stable isotope tracing confirmed upregulation of glucose consumption during osteoblast differentiation and the predominant production of lactate from glucose in mature osteoblasts.^132^ The brisk glucose-to-lactate conversion in the presence of oxygen, known as aerobic glycolysis, was estimated to produce approximately 80% of the energy in mature osteoblasts following differentiation of calvarial preosteoblasts in vitro, a notable increase from 40% in the preosteoblasts.^132^ This shift away from OXPHOS towards glycolysis is not well understood but could potentially relate to concurrent biomineralization in osteoblasts, as a role for mitochondria in mineralization has been long suggested by early investigators.^133^ More recent studies with nano-analytical electron microscopy have further revealed a continuum of calcium phosphate within osteoblast mitochondrial granules, in vesicles cojoining mitochondria and intracellular vesicles that transported materials to the extracellular matrix.^134^ It is however not known at present whether initiation of mineralization in the mitochondria can account for the decrease in mitochondrial respiration. In addition, osteoblasts are known to actively secrete citrate which contributes to the mineral properties of bone.^135–139^ Blocking a major citrate transporter SLC13A5 led to increased conversion of glucose to citrate but reduced OXPHOS.^139^ However, it is not yet known whether increased citrate cataplerosis in mature osteoblasts is responsible for the overall decrease in mitochondrial respiration compared to that in preosteoblasts.

Glucose uptake in osteoblast lineage cells is mainly mediated by members of the GLUT family. RNA-seq in murine calvarial preosteoblast cultures confirmed previous finding of GLUT1 as the predominant transporter and uncovered the expression of additional members including GLUT8 and GLUT10.^132,140^ Besides immediate fueling of glycolysis, the imported glucose may be converted to glycogen for later use, as electron microscopy identified prominent glycogen granules in preosteoblasts that were later reduced in mature osteoblasts, presumably due to increased glycogenolysis.^141^ GLUT1 has been shown to promote osteoblast differentiation by increasing RUNX2 protein levels in one study but appears to be mainly required for supporting the mineralizing activity of osteoblasts in another study.^63,140^ Delineation of individual GLUT contribution in osteoblasts is complicated not only by functional redundancy among the many family members, but also by metabolic plasticity that could involve other energy substrates as discussed below.

In concert with glucose metabolism, amino acid metabolism is also crucial for mineralization osteogenic differentiation. When arginine was introduced to differentiating osteoblasts and pre-osteoblasts at increasing concentrations, IGF1 expression and collagen I synthesis was stimulated.^142^ Arginine can also be converted to nitric oxide that in turn promotes osteoblast differentiation and function via stimulation of glycolysis.^143^ Glutamine was shown to be required for matrix mineralization in osteoblasts.^144^ Energetically, glutamine is converted to a-ketoglutarate to enter the TCA cycle and contribute to energy production.^145^ Glutamine also contributes to redox homeostasis through its contribution to glutathione biosynthesis, improving of survival of osteoblast lineage cells.^146^ Glutamine uptake is primarily mediated by SLC1A5 in osteoblasts under basal conditions about requires SLC7A7 when stimulated by WNT.^147^ Glutamine uptake via SLC1A5 is important for osteoblast differentiation partly by supporting the biosynthesis of non-essential amino acids.^148^ Other amino acids including proline, alanine and asparagine have also been implicated in osteoblast differentiation.^148,149^

Fatty acids have been implicated in osteoblast differentiation and function. Several studies have reported bone as a major site of lipid and fatty acid uptake.^150–152^ Deletion of CPT2 in osteoblasts impaired peak bone accrual in female but not male mice.^151^ However, as RNA-seq detected downregulation of fatty acid β-oxidation genes with calvarial osteoblast differentiation, the dependence on fatty acids as an energy source may be transient in the osteoblast lineage.^132^

Several osteogenic transcription factors have been shown to intersect with cellular metabolism. RUNX2, the master regulator of osteogenesis activates GLUT1 expression to stimulate glucose metabolism in osteoprogenitors.^140^ ATF4 increases the amino acid uptake necessary for collagen synthesis, which is necessary for osteoblast function.^91^ HIF1α overexpression has been shown to stimulate osteoblast differentiation and the osteogenic role is dependent on increased glycolysis.^153^

**Fig. 8:**
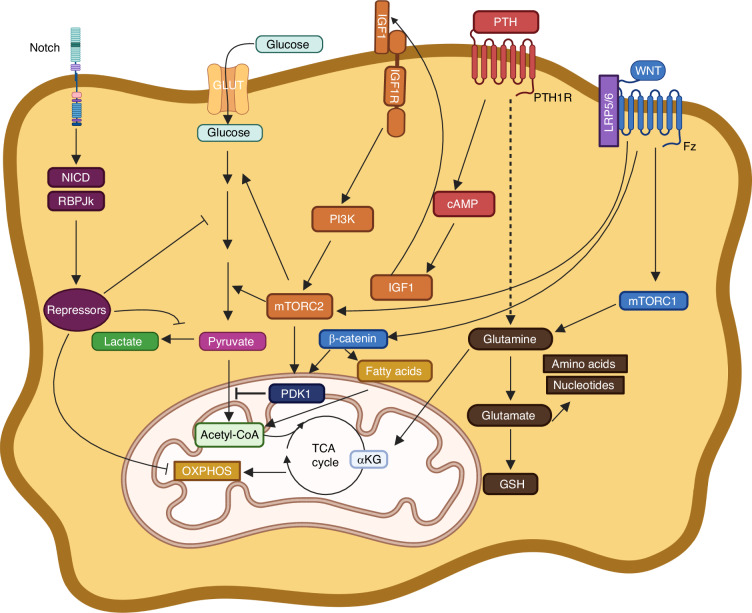
Metabolic reprogramming during osteoblast differentiation by growth factors. GSH: reduced glutathione. α-KG: alpha-ketoglutarate. Dashed line denotes unknown mechanism

Multiple growth factors have also been implicated in metabolic regulation in osteogenic cells (Fig. 8). WNT signaling, a major bone anabolic mechanism, has been shown to activate several bioenergetic pathways in osteoblasts.^154^ WNT signaling increases aerobic glycolysis by activating mTORC2 and AKT, resulting in increased protein abundance of key glycolytic enzymes.^155^ Deletion of Rictor, an essential component of mTORC2, diminished the bone anabolic effect of a sclerostin antibody that boosts WNT signaling in postnatal mice.^156,157^ Moreover, WNT7B overexpression in osteoblasts causes excessive bone formation in the mouse, and the bone overgrowth is dependent on GLUT1 upregulation.^158^ In addition, LGR4, a potentiator of WNT-β-catenin signaling, increases aerobic glycolysis and osteoblast differentiation by activating PDK1 expression.^159,160^ Mechanistically, metabolic reprogramming by WNT has been linked with reduced acetyl-coA production and large scale gene suppression during osteoblast differentiation in vitro.^161^

WNT signaling has also been linked to stimulation of glutamine and fatty acid oxidation in osteoblast lineage cells (Fig. 8). Increased glutamine oxidation in osteoprogenitor cells triggers GCN2-mediated integrated stress response that is necessary for augmenting the biosynthetic capacity in transition to functional osteoblasts.^145^ The stimulation of glutamine consumption by WNT is mediated by mTORC1 independent of β-catenin.^145^ In contrast, WNT enhances fatty acid oxidation via β-catenin which upregulates the expression of multiple enzymes in the pathway.^162^

Parathyroid hormone (PTH) and its therapeutic derivative teriparatide-a mainstay of bone anabolic therapy-have been shown to promote aerobic glycolysis in osteoblasts via the induction of IGF1, which in turn activates PI3K and mTORC2^163^ (Fig. 8). In addition, PTH has been shown to stimulate glutamine catabolism in osteoprogenitors to support nucleotide and amino acid synthesis as well as glutathione production necessary for increased bone anabolism.^164^

Contrary to the bone anabolic signals above, Notch signaling suppresses osteoblastogenesis in vivo.^122,124,165^ Consistent with this role, Notch inhibits the glycolytic pathway in bone marrow mesenchymal progenitors in a RBPjk-dependent manner^166^ (Fig. 8). Thus, both positive and negative effects of multiple intercellular signals on osteoblast differentiation are concordant with their regulation of glycolysis in osteoblast lineage cells.

Few studies have been conducted to examine cellular metabolism in osteocytes. In vivo carbon tracing with labeled glucose identified lactate as the predominant metabolite from glucose in the cortical bone containing mostly osteocytes, thus supporting aerobic glycolysis as a main metabolic pathway in osteocytes like osteoblasts.^132^ On the other hand, bone defects in growth hormone receptor null mice have been linked with reduced mitochondrial membrane potential and impaired respiration in osteocytes.^167^ Future studies are necessary to delineate the relative roles of glycolysis versus OXPHOS in osteocyte bioenergetics and the contributions of various energy substrates to mitochondrial respiration in osteocytes.

### Metabo-epigenetic regulation of osteoblastogenesis

Studies of osteoblast differentiation have begun to link metabolic changes to epigenetic regulations. In mouse bone marrow stromal cell line ST2 cells, osteogenic WNT signaling reduced flux of glucose metabolism in the TCA cycle and diminished the cytosolic-nuclear pool of acetyl-CoA, causing genome-wide decrease in histone acetylation consistent with suppression of gene expression.^161^ More recently, increased lactate production during osteoblast differentiation in MC3T3-E1 cells has been linked with an increase in histone lactylation, including at the promoter region of JUNB, a known regulator of osteoblastogenesis.^168^ Similarly, histone lactylation has been implicated in osteoblast differentiation from C2C12 cells in response to BMP2, although specific target genes were not identified.^169^ In addition, endothelial cell-derived lactate has been shown to increase histone lactylation at the loci of osteoblast marker genes in bone marrow mesenchymal progenitors, and diminution of such metabo-epigenetic crosstalk was further linked with bone loss in female mice following ovariectomy.^170^ In periosteum-derived skeletal progenitors, pharmacological inhibition of Complex III of the electron transport chain increased succinate and 2-hydroxyglutarate levels that inhibited ten-eleven translocation (TET) DNA demethylase activity, causing wide-spread DNA hypermethylation in the genome.^171^ However, future studies are necessary to determine whether TET activity is regulated by metabolite changes during osteoblast differentiation and how it affects the differentiation process.

## Osteoclasts

### Growth factors and nuclear regulators in osteoclast differentiation

**Fig. 9:**
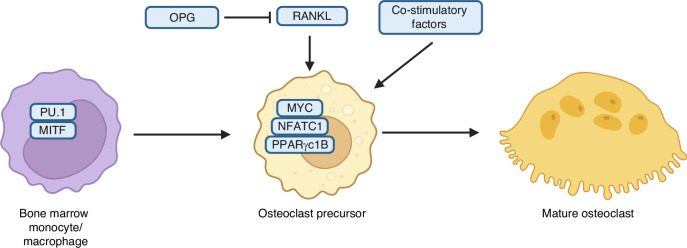
Major transcription factors and cytokines regulating osteoclast differentiation. See text for details and references

Osteoclasts, the cells responsible for bone resorption, are giant multinuclear cells derived from either erythromyeloid progenitors in the yolk sac early in life or circulating monocytic lineage cells descended from bone marrow hematopoietic stem cells in adult animals.^172^ The earliest events of osteoclastogenesis are mediated by the transcription factors PU.1 in collaboration with the MITF family of proteins^173,174^ (Fig. 9). The primary osteoclastogenic signal RANKL (receptor activator of nuclear factor kappa B ligand) via RANK receptor activates multiple transcription factors including nuclear factor kappa B (NF-kB), CEBPα and AP1 factors (c-fos and c-Jun), all of which are required for the initial induction of NFATC1, the master transcription factor for osteoclastogenesis.^175–177^ Transcription factor MYC has also been shown to act downstream of RANKL signaling to induce NFATC1 transcription.^178^ Nuclear localization of NFATC1 is promoted by increased intracellular Ca^2+^ levels in response to activation of co-stimulatory receptors, which together with RANKL signaling is required for osteoclastogenesis.^179^ Furthermore, NFATC1 induces the expression of transcription repressor BLIMP1 which in turn suppresses the transcription of transcription factors IRF8 and MafB which themselves are suppressor of NFATC1 in osteoclast progenitors.^180–182^ Finally, expression of BLIMP1 is negatively regulated by the transcription factor RBPJk.^183^ Thus, osteoclast differentiation is controlled by a myriad of transcription factors acting in coordination with the central regulator NFATC1.

Cytokines, a subset of growth factors, are critical for osteoclastogenesis. Colony-stimulating factor 1 (CSF1), also known as MCSF, signal thorough its receptor CSF1R (also known as CD115) on monocytic cells to promote cell proliferation and to induce the expression of RANK, the receptor for RANKL.^184^ As noted above, RANKL-RANK signaling together with co-stimulation of receptors associated with ITAM (immunoreceptor tyrosine-based activation motif) proteins is required to express and activate NFATC1 (Fig. 9). Under inflammatory conditions, many cytokines including TNFα and several interleukin proteins have been shown to induce osteoclastogenesis directly.^185^ RANKL is produced both as a transmembrane protein and in a soluble form but the membrane-bound form appears to be responsible for the majority of osteoclast formation under normal conditions whereas the soluble form was found to promote cancer cell metastasis to bone in mouse models.^186,187^ Both CXCL12 abundant reticular (CAR) cells in the bone marrow and osteocytes have been shown to be major sources of RANKL supporting osteoclastogenesis.^188–191^ The osteoclastogenic activity of RANKL is counterbalanced by the secreted decoy receptor OPG that prevents RANKL from interacting with RANK in osteoclast progenitors. Tissue-specific deletion studies have demonstrated that locally produced OPG by osteoblasts is mainly responsible for suppressing osteoclastogenesis in bone.^192,193^ Overall, the RANKL:OPG ratio in local microenvironment is central to the control of osteoclast formation and bone resorption in both normal and diseased conditions.

MCSF and RANKL also promote osteoclast survival and activity thorough activation of mTOR (mammalian target of rapamycin)/S6 kinase (mTORC1) signaling, which is a central nutrient-sensing mechanism in mammalian cells.^194^ Moreover, mTORC1 has been implicated in cytoplasm growth through increased protein synthesis in nutrient replete conditions whereas mTORC2 activity promoted cell fusion to form multinucleated osteoclasts.^195^ Thus, mTOR signaling appears to promote distinct steps of osteoclastogenesis in response to bioenergetic conditions.

### Osteoclast metabolic regulation

As professional bone-resorbing cells, osteoclasts secrete copious amounts of acids and proteolytic enzymes and require a large supply of energy. Multiple studies have demonstrated de novo mitochondrial biogenesis during osteoclastogenesis and that OXPHOS is the main bioenergetic mechanism in the process.^196–198^ Deletion of NDUFS4, a component of Complex I of the mitochondrial electron transport chain, impaired osteoclast differentiation and function in the mouse.^199^ In addition, deletion of the mitochondrial transcription factor TFAM in mature osteoclasts of the mouse reduced intracellular ATP levels and accelerated osteoclast apoptosis.^200^ Besides ATP production, mitochondrial respiration also produces ROS that has been shown to stimulate osteoclastogenesis.^201^ The studies collectively support critical roles of mitochondrial respiration in osteoclast formation.

Glucose is a major energy substrate supporting both osteoclast differentiation and function in chickens, mice, and humans.^196,197,202,203^ Besides fueling OXPHOS, glucose metabolism via aerobic glycolysis also increases with and is necessary for osteoclast differentiation.^197,204^ Suppression of both LDHA and LDHB, which reduces both aerobic glycolysis and mitochondrial respiration, has been shown to reduce NFATC1 expression and osteoclast differentiation.^204^ Moreover, deletion of GLUT1 in osteoclast progenitors, diminished aerobic glycolysis without compromising OXPHOS, but nonetheless impaired osteoclast differentiation in vitro, thus further supporting the role of aerobic glycolysis.^197^ On the other hand, blocking pyruvate entry into the mitochondria by deleting MPC1 also impaired osteoclastogenesis in vitro and caused osteopetrosis in female mice, thus supporting the importance of glucose in fueling OXPHOS.^205^ Finally, there is evidence that human mature osteoclasts rely more heavily on glycolysis than OXPHOS to support resorptive activity.^196^ Thus, de novo mitochondria biogenesis and increased energy production via OXPHOS are hallmarks of osteoclast differentiation, but aerobic glycolysis also plays important roles both during differentiation and in support of bone resorption by mature osteoclasts.

Other energy substrates have also been implicated in osteoclast differentiation. Besides glucose, consumption of amino acids and long-chain fatty acids increases with osteoclast differentiation.^205^ Deletion of CPT1A, which is required for β-oxidation of long-chain fatty acids in the mitochondria, impaired osteoclasts in vitro, and disrupted bone homeostasis specifically in female mice.^205^ Contrary to long-chain fatty acids, short-chain fatty acids including propionate and butyrate inhibited osteoclastogenesis by suppressing OXPHOS in osteoclast progenitors.^206^ Glutamine uptake was required for osteoclastogenesis in vitro, where glutamine can be replaced by dimethyl α-ketoglutarate, a membrane permeable analog of α-ketoglutarate.^203^ However, a specific requirement for glutamine in energy production via OXPHOS during osteoclast differentiation remains to be demonstrated. Finally, environmental arginine is required for RANKL-induced osteoclast differentiation independent of mTORC1 but instead through its role in increased mitochondrial respiration.^207^

**Fig. 10:**
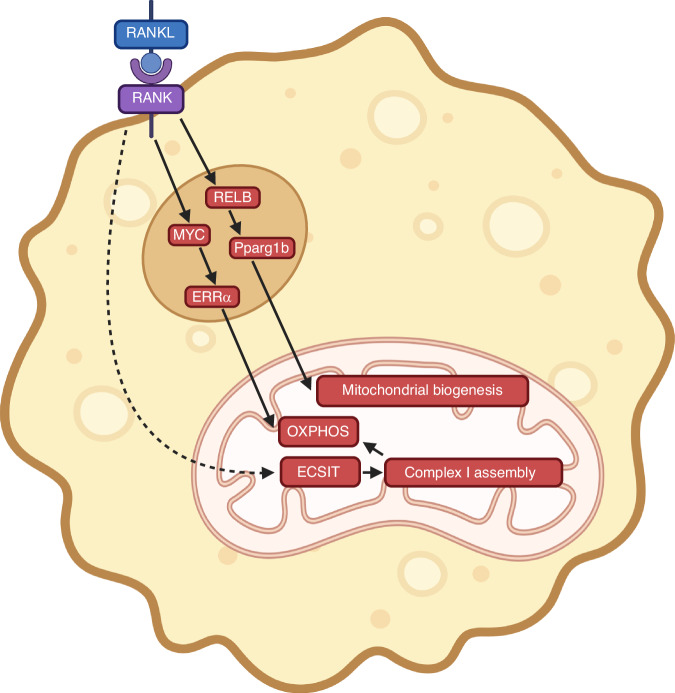
Metabolic reprogramming by RANKL during osteoclast differentiation. Dashed line denotes unknown mechanism

RANKL directs metabolic reprogramming towards OXPHOS during osteoclast differentiation through multiple mechanisms (Fig. 10). RANKL stimulates mitochondrial biogenesis through alternative NF-kB signaling and upregulation of PPARGC1B in osteoclast precursors.^198,208,209^ RANKL also conducts metabolic reprogramming through the transcription factor MYC which in turn induces the transcription of ERRα (estrogen receptor–related receptor α), upregulating genes involved in the TCA cycle and OXPHOS.^210^ Both metabolic reprogramming by MYC and activation of osteoclast identity genes by NFATC1 are necessary for completing osteoclastogenesis in response to RANKL.^210^ RANKL has been further suggested to promote the translocation of ECSIT (evolutionarily conserved signaling intermediate in Toll pathways) to the mitochondria where it facilitates Complex I assembly and thus promotes mitochondrial respiration, but the exact mechanism for the mitochondrial translocation remains to be elucidated.^211^ Thus, RANKL stimulates mitochondrial respiration during osteoclast differentiation through both transcriptional regulation and other mechanisms.

### Metabo-epigenetic regulation of osteoclastogenesis

In osteoclastogenesis, α-ketoglutarate has been shown to function as a co-factor for histone demethylases to regulate differentiation. In a recent study, α-ketoglutarate generated from a transaminase reaction in the serine biosynthesis pathway stimulated osteoclast differentiation by promoting histone demethylase activities that remove repressive histone methylation at the promoter of NFATC1.^212^ In an earlier study, exogenously applied α-ketoglutarate in the form of dimethyl-α-ketoglutarate was shown to suppress osteoclast differentiation via de-repressing of SLC7A11 expression, resulting in increased glutathione synthesis and reduced ROS levels in osteoclast progenitors.^213^ Thus, α-ketoglutarate as a co-factor for histone demethylases likely has many target genes whose regulation may be sensitive to different concentrations of α-ketoglutarate and other cellular contexts. As α-ketoglutarate can also be derived from glutamine, it will be of interest to determine whether glutamine also exerts epigenetic regulation of osteoclastogenesis via α-ketoglutarate. Therefore, much is yet to be explored about the nature and extent of metabo-epigenetic regulation in osteoclastogenesis.

## Dysregulation of bone cell metabolism in diabetes

Diabetes has been most extensively studied among the systemic metabolic disorders linked with skeletal comorbidities. Recent studies in mouse models have provided evidence that cell-intrinsic metabolic defects in osteoblasts are a driving force in diabetic osteopenia. In particular, the glycolysis pathway, chiefly responsible for energy production in osteoblasts, is impaired in the bones of mouse models for either type I or type II diabetes.^214,215^ More importantly, forced activation of glycolysis specifically in the osteoblast lineage was sufficient to ameliorate bone loss in the diabetic mice. The exact mechanisms responsible for impaired osteoblast glycolysis in diabetes remain to be fully elucidated but likely involve both defective insulin signaling and hyperglycemia toxicity.^214,216,217^ The involvement of insulin signaling likely differs between bone compartments as insulin receptor deletion specifically impaired cortical bone accrual whereas insulin therapy failed to restore trabecular bone formation in a type I diabetes mouse model.^214,218^ In addition, lipotoxicity in diabetes could contribute to insulin insensitivity in osteoblasts, as previously shown in mice on a high fat diet.^219^ Besides osteoblasts, osteoclasts are also affected resulting in impaired bone resorption in diabetes, but specific effects on metabolic pathways in osteoclasts are yet to be explored.^220^ Diabetes is generally associated with increased risk and progression for osteoarthritis, which has in turn be linked with impaired glucose metabolism in articular cartilage.^68,221^ Overall, future studies are expected to reveal the full spectrum of metabolic defects in bone cells associated with diabetes along with other systemic metabolic diseases.

## Conclusions and perspective

Research in the past decade or so has provided unprecedented insights into the metabolic features and regulation of various skeletal cells. Chondrocytes, consistent with their native environment of avascular cartilage with limited access to oxygen, derives most energy from glycolysis, but TCA metabolism and mitochondrial respiration are necessary for proper progression to chondrocyte hypertrophy. Besides glucose, amino acids including glutamine contribute to chondrocyte proliferation and matrix protein synthesis. The master chondrogenic transcription factor Sox9 on the one hand promotes glutamine utilization and on the other hand suppresses fatty acid oxidation, thus orchestrating major metabolic rewiring necessary for chondrogenesis. In addition, multiple chondrogenic growth factors including Bmp, CCN2 and Igf2 have been shown to reprogram cellular metabolism to favor glycolysis in chondrocytes. Like chondrocytes, osteoblasts also rely on glycolysis for most energy production even though bone tissues are generally well vascularized. The exact mechanism for the bioenergetic switch from mainly OXPHOS in precursors to aerobic glycolysis in osteoblasts is unclear at present and awaits to be elucidated in the future. Glutamine not only contributes to TCA cycle metabolism for energy production in osteoprogenitors but also maintains redox balance through glutathione production, whereas fatty acid oxidation may transiently contribute to bioenergetics during osteoblast differentiation. The osteogenic transcription factors Runx2 and Atf4 promote glucose and amino acids uptake, respectively, to meet the increased demand for energy and building blocks in osteoblasts, whereas bone anabolic signals such as Wnt and teriparatide augment energy production from glucose, fatty acids and glutamine in osteoblast lineage cells. Different from chondrocytes and osteoblasts, osteoclasts are highly enriched for mitochondria and derive most energy through OXPHOS although aerobic glycolysis also supports both differentiation and the resorptive activity of osteoclasts. Multiple energy substrates including glucose, fatty acids and glutamine likely fuel OXPHOS to support osteoclast differentiation and function. The central osteoclastogenic signal RANKL stimulates mitochondrial biogenesis and OXPHOS through multiple mechanisms. Collectively, the findings support the theme that beyond inducing cell identity genes, both transcription factors and growth factors also reprogram cellular metabolism to accommodate the bioenergetic and biosynthetic changes integral to cell differentiation.

Several studies to date have linked changes in specific metabolites including acetyl-coA, lactate and α-ketoglutarate to expression of identity genes in chondrocytes, osteoblasts or osteoclasts due to histone or DNA modification. However, epigenetic regulation by metabolites is inherently broad-based and impacts the expression of many genes. Future studies are necessary not only to identify genome-wide histone and DNA modifications associated with specific metabolic changes but also to pinpoint pertinent target genes whose epigenetic regulation impacts cell differentiation and function.

There remain major technological barriers in studying skeletal cell metabolism in vivo. Gene or protein expression profiles are suggestive but not direct representation of metabolic activities. In vivo stable isotope tracing of energy substrates in bone is quantitative but does not provide spatial resolution or cellular specificity in the tissue.^132^ Future use of genetic reporters for specific metabolites in vivo or nano-scale metabolic imaging will greatly advance the field.^222,223^

Elucidating the metabolic profiles for skeletal cells under both normal and pathological conditions has important translational and clinical implications. On the one hand, future development of quantitative imaging techniques for detecting metabolic changes in bone or cartilage may provide early diagnosis of osteoporosis or osteoarthritis before morphological defects become apparent in conventional exams. On the other hand, development of metabolism-based drugs may lead to a new class of therapy for either treating chronic skeletal conditions or enhancing bone injury repair.
